# The use of Polidori's plasticity and activity charts in classifying some residual lateritic soils from Nigeria

**DOI:** 10.1016/j.heliyon.2021.e07713

**Published:** 2021-08-03

**Authors:** Lekan Olatayo Afolagboye, Abel Ojo Talabi, Olubunmi Oluwadare Owoyemi

**Affiliations:** a Department of Geology, Ekiti State University, Ado-Ekiti, Nigeria; b Department of Geology, Kwara State University, Malete, Nigeria

**Keywords:** Atterberg limits, Clay fractions, Soil classification, Plasticity charts

## Abstract

Over the years, Casagrande plasticity chart is mainly used to classify fine grain soils. However, the use of the plasticity chart has been questioned recently and this has led to the development of a new plasticity chart. Polidori in 2007 and 2009, respectively, developed the new plasticity and activity charts using the Atterberg's limits of pure clays (montmorillonite and kaolinite clay minerals) and their mixture with fine silica sand in different proportions. The applicability of Polidori's charts was evaluated using some residual lateritic soils from Nigeria. On the Casagrande's plasticity chart, the lateritic soils mostly plot above the A-line in the zone designated as clay and classified as either CL or CH. However, on the Polidori's plasticity chart, the lateritic soils classified as CL or CH, whereas on Casagrande's plasticity chart they are classified as ML or MH and vice versa. The classifications obtained from Polidori's plasticity chart are predominantly in agreement with the main soil fractions or component of the soils. This is different from the classification obtained from Casagrande's plasticity chart where lateritic soils with lower clay fractions than their silt/sand fractions are classified as clayey soils. Polidori's activity chart shows that lateritic soils that lie in the same plastic zone may show different behavior due to the different properties of the clay minerals in the soils. In cases where the lateritic soils lie in the zone that is not corresponding to their clay contents on the Polidori's plasticity chart, we presume that other factors apart from those stated by Polidori might also be responsible. Although the use of Polidori's plasticity chart gives a fair classification of the lateritic soils, nevertheless the peculiarity of residual soils such as the in situ structure that influenced the properties of the soils and properties developed due to weathering effects must be taken into consideration as well.

## 1 Introduction

Scientists and engineers use soil classification systems to predict soil behavior and response. According to Smith (2014), soil classification allows engineers to allocate a soil to a specified number of groups based on the soils material properties. For decades, soil classification systems that use plasticity and particle size (grading) to placed soils into different behavioral groups have been in existence (Duarte et al., 2018; McCarthy, 2014). The Unified Soil Classification System (ASTM, 2011), The American Association of State Highway Transportation Officials (AASHTO, 1991) classification, British Standards (BSI, 1990) and Australian Standards (AS) are few examples of soil classification systems currently in use. The aforementioned classification systems generally classify soils as fine and coarse-grained soils (cohesive soils and non-cohesive).

According to ASTM and AASHTO standards, soils in which 50 % and 35 % (or less), respectively, are finer than the No. 200 sieve (0.075mm) are termed coarse-grained soils. Coarse grained soils are mainly classify using the particle size distribution. On the other hand, fine grained soils (soils that possess high percentage (>35 or 50 %) of fractions finer than No. 200 sieve) are classified using the plasticity characteristics of the <0.425 mm soil fraction. The plasticity of fine-grained soils can be obtained using the Casagrande (1948) plasticity chart where the Plasticity Index (I_P_) of the soil are plotted against the Liquid Limit (W_L_) of the soil. The A-line in the chart allows us to differentiate between the inorganic clays and inorganic silts or organic soils located above and below the A-line, respectively. Inorganic clays and both inorganic silts and organic soil that respectively lie above and below the A-line are said to have low or high plasticity if their W_L_ is less or more than 50 % according to ASTM standard D2487 (ASTM 2011).

Despite the popularity of the classification system, some researchers have pointed out the shortcomings of the plasticity chart (Fourie et al., 2012; Jang and Santamarina, 2015; Polidori, 2003) and have led to the proposal of a new plasticity chart (Polidori, 2003, 2007, 2009). Polidori (2003) questioned the reason why inorganic soils with clay fractions lower than sand and/or silt fraction plotted on the Casagrande's chart plot above the A-line (clay zone) and why pure kaolinite plots below the A-line (silt zone) on the plasticity chart. In addition, the author also questioned the significance of the distance, from the A-lines, of the different points plotted on Casagrande's plasticity chart. One of the points raised by Jang and Santamarina (2015) is that the weight of the coarse non-plastic fraction determined the classification of soil mixtures made up of non-plastic and plastic paticles while the classification of high-plasticity fines is determined by sediment hydraulic and mechanical properties.

In 2007, Polidori developed a new plasticity chart that is significantly different to the previously known Casagrande's plasticity chart. Polidori argued that the Casagrande's plasticity chart was developed without looking at the effect of the clay fraction (CF) of the soil and this led to the difference in the position of clay and silt zones on the two plasticity charts. Polidori developed his plasticity chart using the Atterberg limits of pure kaolinite, pure montmorillonite and their mixture with fine silica sand in various proportions. Polidori (2007) observed that W_L_ and clay content of a soil are the most effective parameters to evaluate soil properties and based on this observation Polidori (2009) proposed a new activity chart that classifies soils or soil fractions <425μm using the Atterberg limits and amount clay minerals rather than their plasticity.

Laterite and lateritic soils, an example of tropical residual soils, may have different engineering and index characteristics depending on the mineralogy of the parent rock, weathering intensity, rainfall, and temperature. These properties vary with spatial location as well as depth (Adeyemi and Wahab, 2008; Ogunsanwo, 1986). Studies have also shown that field behavior/performance of lateritic soils with similar plasticity characteristics could vary significantly (Adeyemi and Oyeyemi, 2000). In addition, classification of these soils on the Casagrande plasticity chart is often different to the physical observation of hand specimens or not in agreement with the main fractions (silt or clay) defined by particle size distribution of the soil.

This paper examines the distinction between proposed Polidori's and Casagrande's plasticity charts to see if the new plasticity chart will give a soil classification similar to physical observation of hand specimens of the lateritic soils or it will be in agreement with the main components defined by grain size distribution. To achieve these aims, the results of the consistency limits and grain size analysis of different lateritic soils from different parts of Nigeria, whose values have been reported in the literature, were used to evaluate the applicability of Polidori's modified plasticity and activity charts. The parent rocks from which the lateritic soils are derived, liquid limit, plasticity index, Skempton activity, percentage gravel/sand/silt/clay fractions, clay mineralogy (if reported) of the lateritic soil as well as a method of testing adopted by the authors are shown in supplementary file (Table S1). The clay fractions of the lateritic soils mostly composed of kaolinite clay minerals based on the X-ray diffraction analysis.

## 2 Plasticity charts

### 2.1 Polidori's plasticity chart

**Figure 1:**
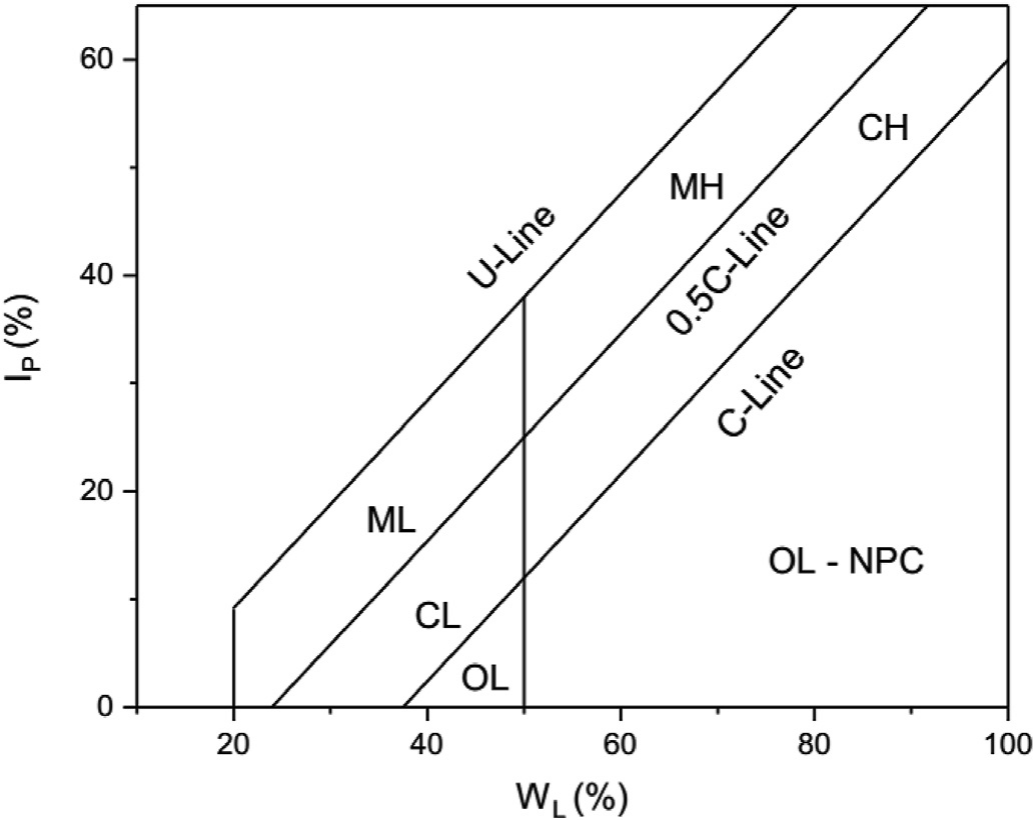
Polidori's plasticity chart (Polidori 2007). CL: Clay zone (CF ≥ 50 %) with low plasticity; CH: Clay zone with high plasticity; ML: Silt zone (CF < 50 %) with low plasticity; MH: Silt zone with high plasticity; OL: Organic soil with low plasticity; OH: Organic soil with high plasticity; NPC: soils containing non-platey clay minerals.

Polidori in 2007 developed a new plasticity chart. His aim was to develop a plasticity chart for soil or soil fractions with size <0.425 mm that would reflect the percentage clay fraction for accurate classification. Polidori controlled the clay fraction of the soils by adding fine silica sand to soils with 100 % clay fractions (in this case pure kaolinite and montmorillonite clay minerals). Figure 1 shows the Polidori's modified plasticity chart (Polidori, 2007).

In the plasticity chart, Polidori defined two new lines called C – line and 0.5C – line. He argued that the plasticity index (PI) – Liquid Limit (W_L_) values of inorganic soil (with platey clay minerals) that contain less than 100 % clay fractions would plot above the C – line. In addition, the distance of the plotted points (PI – W_L_ values) from the C-line on the chart is inversely proportional to the CF percentage of the inorganic soil. The 0.5C – line separates inorganic soil that contains <50 % CF located above the line (termed silt) and inorganic soil that contains ≥50 % CF located below the line (termed clay).

In both plasticity charts, the U – line represents the upper limit of most soils. Polidori (2007) gave the equations of C-line (Eq. (1)), 0.5C – line (Eq. (2)), and U – line (Eq. (3)), respectively as

(1) I_P_ = 0.96W_L_ – 36

(2) I_P_ = 0.96W_L_ – 23

(3) I_P_ = 0.96W_L_ – 10

Polidori subdivided the silt and clay zones into high (H) or low (L) plasticity zone when the W_L_ value is greater or less than 50 %, respectively as stipulated by ASTM standard D2487 (ASTM 2011). Polidori (2009), however, cautioned that this plasticity chart might not be suitable for inorganic residual soils comprising non-platey clay minerals because they have properties that are remarkably different from residual organic soils containing platey clay minerals (Mitchell and Soga, 2005; Wesley, 1992). This may make soils with non-platey clay minerals to fall above or below the C-line depending on the prevailing characteristics.

### 2.2 Casagrande's plasticity chart

**Figure 2:**
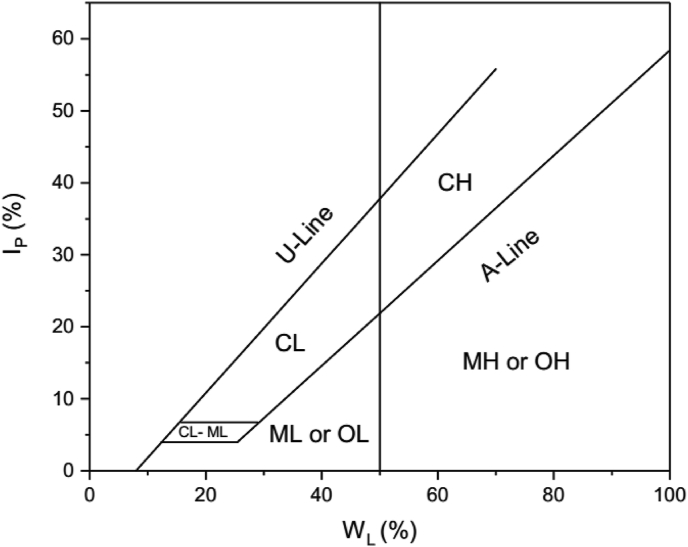
Casagrande's plasticity chart (Casagrande, 1948). CL: Inorganic clays of low plasticity, CH: Inorganic clays of high plasticity; ML: Inorganic silts of low compressibility; MH: Inorganic silts of high compressibility; OH: Organic clay of high compressibility; OL: Organic clay of low compressibility.

In 1948, Casagrande proposed the relationship between I_P_ and W_L_ using a wide range of natural soils. From this relationship, he produced the Casagrande's plasticity chart (Figure 2) where the A-line (I_P_ = 0.73(W_L_ – 20)) in the chart separates the inorganic silts from clays. In this chart, organic silts and clays may plot below or above the A-line. The U-line denotes the upper limit of the relationship between I_P_ and W_L_ for any known soil. The silt and clay zones are divided into high (H) or low (L) plasticity zone when the W_L_ value is greater or less than 50 %, respectively. Comparing the two plasticity charts (Figure 1 and Figure 2), one can see that there are remarkable differences between the two plasticity charts.

## 3 Polidori's activity charts

**Figure 3:**
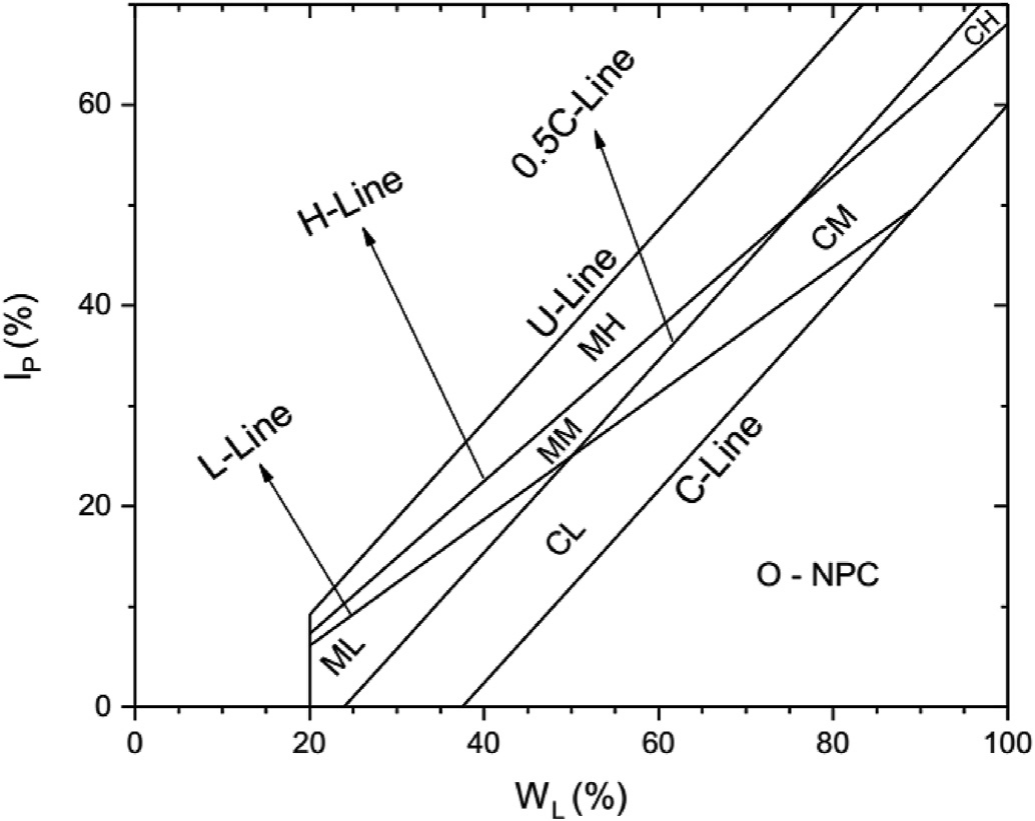
Polidori Activity Chart (Polidori, 2009). H-Line and L-line: lines on which I_P_ vs W_L_ values of inorganic soil with 1.0 and 0.5 values of activity, respectively, should lie; CL, CM, CH: groups of clays (CF ≥ 50 %) with low, medium and high activity, respectively; ML, MM, MH: groups of silts (CF > 50 %) with low, medium and high activity, respectively; O: organic soils; NPC: soil with non-platey clay minerals.

In inorganic soils that contain a reasonable amount of platey clay minerals, Polidori (2007) observed the relationship between W_L_, plastic limit (W_P_), I_P_, activity (A), and CF. Based on this interdependence, Polidori (2009) argued that inorganic soils (with fraction >0.425 mm) containing platey clay minerals can be classified with the aid of their Atterberg limits and clay percentage on the basis of their Activity (A) instead of their plasticity characteristics as a function of W_L_ as it is being used currently by the different standards, and came up with another chart called “Activity Chart” (Figure 3).

In addition to C-line and 0.5C-line, the activity chart contains additionally two new lines (L-line and H-line) that define three zones with different values of activity. The L-line and H-line (A = 0.5 and A = 1.0 respectively) subdivide the silt and clay zone into groups: low activity (L), medium activity (M), and high activity (H) located below, between and above the two boundaries (L-line and H-line). He replaces the term inactive in Skempton activity classification with low activity in the new activity chart. Polidori (2009) gave the equations of H-line (Eq. (4)) and L-line (Eq. (5)), respectively as

(4) I_P_ = 0.76W_L_ – 7.9

(5) I_P_ = 0.63W_L_ – 6.5

Polidori argued, “Soil containing mostly kaolinite and montmorillonite clay minerals (regardless of the clay percentage they contain) should lie in the low activity zone and in the high activity zone, respectively”.

## 4 Comparison between Polidori and Casagrande charts of the lateritic soils

The soils used in this study are lateritic soils, from different parent rocks, whose values of the consistency limits and grain size analysis were reported in the literature. The values of these parameters as reported by the authors are shown in Table 1. Almost all soils have high sand and silt fractions (over 50 % combined). Although, the particle size distributions of lateritic soils presented by Ogunsanwo (1986) have a high percentage of clay fractions compared with the combined sand and silt fractions.

**Table 1:** Index properties and plasticity classifications of the lateritic soils.

No	W_L_ (%)	I_p_ (%)	Grain Size (%)				Plasticity Classification	
			G	S	M	C	Casa (1948)	Pol (2007)
1	65.2	34.3	-	13	24	63	CH	CH
2	59.5	34.6	-	14	29	57	CH	CH
3	64	31.4	-	18	22	60	MH	CH
4	64.7	35.9	-	13	25	62	CH	CH
5	68.8	35.6	-	15	25	60	MH	CH
6	66.3	38.3	-	18	22	60	CH	CH
7	67.7	33.9	-	18	25	57	MH	CH
8	35.2	11.6	2	46	40	12	CL	ML
9	42.2	6.5	0	35	60	5	ML	CL
10	49.5	28.4	2	48	15	35	CL	ML
11	47.9	28.2	12	35	24	29	CL	ML
12	55.5	30.1	3	23	36	38	CH	CH
13	58	31.3	18	40	32	10	CH	CH
14	47.9	30.3	7	45	38	10	CL	ML
15	50	23.7	3	49	39	9	CL	CL
16	44.3	23.5	10	42	41	7	CL	ML
17	60.2	28.5	4	22	62	12	MH	CH
18	57	22.5	3	29	55	13	MH	CH
19	58.1	26.3	1	37	49	13	MH	CH
20	56.8	19.5	6	34	32	28	MH	CH
21	36.7	9.3	53	29	13	5	ML	CL
22	46.1	16.1	12	40	30	18	ML	CL
23	43.7	9.4	8	44	27	21	ML	CL
24	44.7	13.6	7	44	28	21	ML	CL
25	49.9	26.4	3	57	19	21	CL	ML
26	48.5	25.3	9	48	22	21	CL	ML
27	51.4	31.7	1	20	45	34	CH	MH
28	45	16	12.5	48	32.5	7	ML	CL
29	42	14	13	35	32	20	ML	CL
30	43	20	0.3	37	29.7	33	CL	ML
31	41	19	0.6	29	37.4	33	CL	ML
32	40	18	3.8	37	26.2	33	CL	ML
33	46	26.4	19	26.2	41.8	13	CL	ML
34	46	26.3	18	29.2	39.8	13	CL	ML
35	49.5	27.3	25	22.7	46.8	5.5	CL	ML
36	54.5	28.5	24	23	47	6	CH	CH
37	39	15.5	20	22.5	49.5	8	CL	ML
38	41.6	16	18	24.9	49.1	8	CL	CL
39	44.5	21.9	23	20.8	53.2	3	CL	ML
40	39	23.3	22	20.8	54.2	3	CL	ML
41	46	17.08	3	42	24	31	ML	CL
42	45	16.66	4	50	29	17	ML	CL
43	46	18.95	6	41	21	32	ML	CL
44	44	19.39	5	48	24	23	CL	CL
45	37	13.89	6	49	24	21	CL	ML
46	42	13.87	6	48	24	22	ML	CL
47	38	15.34	8	52	15	25	CL	ML
48	38	16.7	14	41	20	25	CL	ML
49	36	11.47	6	48	24	22	ML	CL
50	35	9.47	8	47	27	18	ML	CL
51	44	21.4	4	49	22	25	CL	ML
52	42	16.85	2	57	26	15	CL	ML
53	42	21	4	51	25	20	CL	ML
54	44	18.06	7	51	20	22	CL	CL
55	34	14.19	10	57	17	16	CL	ML
56	36	14.87	10	50	23	17	CL	ML
57	42	19	3	60	22	15	CL	ML
58	38	17.82	2	62	23	13	CL	ML
59	42	18.01	3	67	16	14	CL	ML
60	47	20.16	8	57	16	19	CL	CL
61	33	19	8	27	57	8	CL	ML
62	44	26	8	35	45	12	CL	ML
63	32	12	14	43	36	7	CL	ML
64	47.2	19.1	3	37	46	17	ML	CL
65	40.1	19.4	2	35	47	16	CL	ML
66	39	19.1	3	37	45	15	CL	ML
67	42	18.9	2	36	47	15	CL	ML
68	36.2	16.2	3	37	44	16	CL	ML
69	39.1	15.2	3	35	45	17	CL	ML
70	41.8	16.8	3	36	45	16	CL	ML
71	40	17.5	2	38	42	18	CL	ML
72	52.2	23.1	3	32	49	16	MH	CH
73	55.3	20.5	3	31	48	18	MH	CH
74	51.1	21.6	2	36	45	17	MH	CH
75	46	19.5	2	33	50	15	CL	CL

G: Gravel, S: Sand, M: Silt, C: Clay, Casa: Casagrande, Pol: Polidori, 1–7: Ogunsanwo (1986), 8–12: Ogunsanwo (1988), 13–24: Adeyemi (1995), 25–27: Ogunsanwo (1996), 28–29: Adeyemi and Oyeyemi (2000), 30–32: Osinubi and Nwaiwu (2006), 33–40: Adeyemi and Wahab (2008), 41–60: Badmus (2010), 61–63: Oyediran and Okosun (2013), 64–75: Adeyemi et al. (2015).

**Figure 4:**
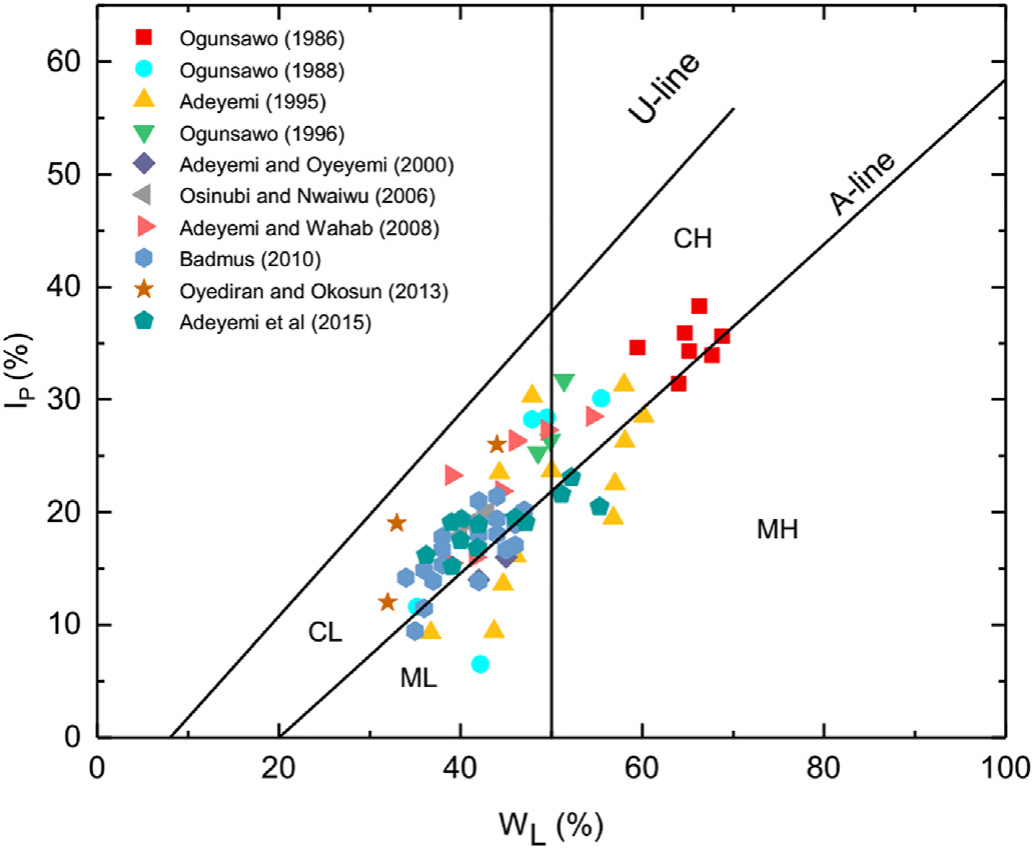
Casagrande (1948) plasticity classification of the lateritic soils. CL: Inorganic clays of low plasticity, CH: Inorganic clays of high plasticity; ML: Inorganic silts of low compressibility; MH: Inorganic silts of high compressibility; OH: Organic clay of high compressibility; OL: Organic clay of low compressibility.

On the Casagrande classification chart (Figure 4, Table 1), most of the soils plot in the clay zone (above the A-line) and classified as either CL or CH soils. Similar to the observation of Polidori (2003), lateritic soils with lower clay fractions than their silt/sand fractions are mostly classified as CH or CL soils. In addition, some of these classifications are also different from physical observation of hand-specimens and not the same with the main component or fractions defined by particle size distribution of the soil. Adeyemi and Wahab (2008) observed that most of the lateritic soils (in their study) are generally sandy silty lateritic soils, but are mostly classified as CL soils on Casagrande's plasticity chart. In addition, the textures of lateritic soils studied by Ogunsanwo (1988) are mostly sandy, but they are also classified as CL or CH soils.

**Figure 5:**
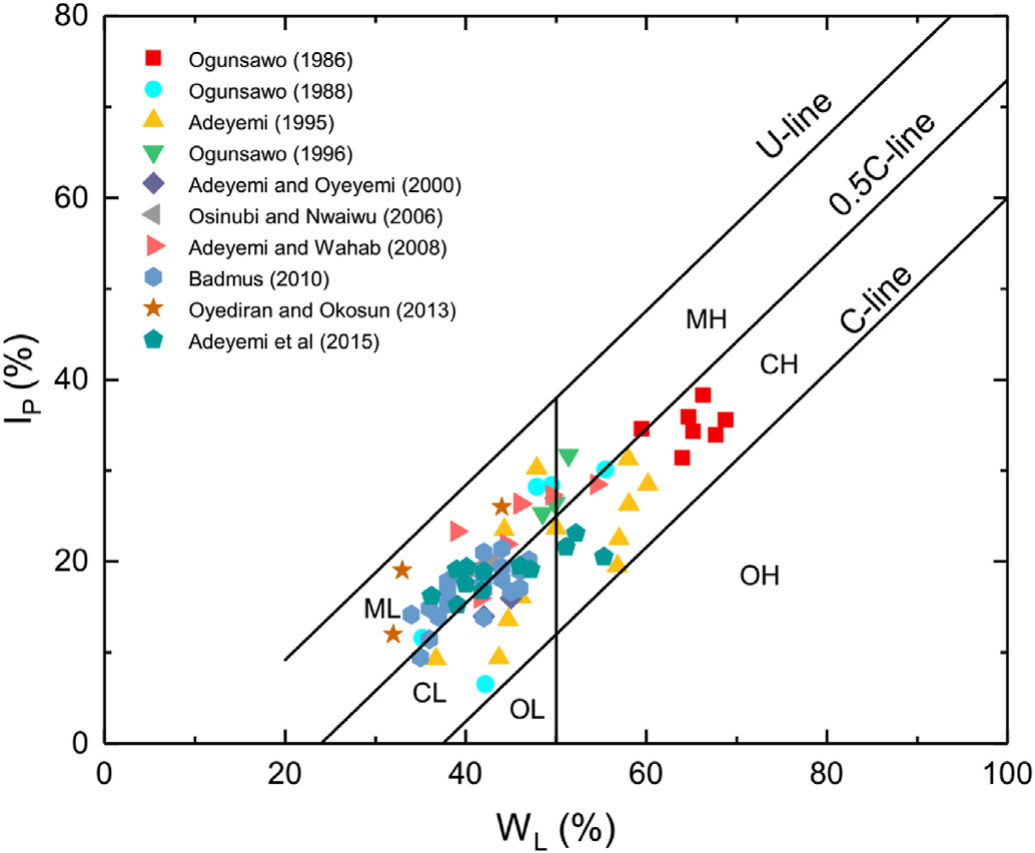
Polidori (2007) plasticity classification of the lateritic soils. CL: Clay zone (CF ≥ 50 %) with low plasticity; CH: Clay zone with high plasticity; ML: Silt zone (CF < 50 %) with low plasticity; MH: Silt zone with high plasticity; OL: Organic soil with low plasticity; OH: Organic soil with high plasticity; NPC: soils containing non-platey clay minerals.

On the modified Polidori classification chart (Figure 5), all the soils lie above the C-line; which imply inorganic soils. The soils are classified as CL, CH, ML or MH based on the modified Polidori classification chart (Figure 5, Table 1). However, lateritic soils classified as CL or CH on the Casagrande's plasticity chart are classified as ML or MH on the modified Poridori's plasticity chart (and vice-versa). In some cases, the lateritic soils are classified as CL or CH on both plasticity charts. According to Polidori (2003), the new modified plasticity chart should produce a classification that will be roughly the same with the main fraction (clay or silt) as defined by the different standards (e.g. British Standard) for the grain size distribution of soil fractions <0.425 mm. However, there are cases where the lateritic soils plot in clay or silt zone when their clay contents are less or greater than 50 %, respectively. Data (or part of data), such as those presented by various authors (Adeyemi, 1995; Adeyemi et al., 2015; Adeyemi and Oyeyemi, 2000; Badmus, 2010), with a significant amount of coarse grain particles (i.e. high silt particles and all plot in clay zones) have this kind of problem.

Polidori (2003) attributes this exception to the problems associated with the determination of the plastic limit of the soil (Temyingyong et al., 2002; Whyte, 1982) and soil samples with a clay fraction of 34–49 %. To further explain the latter factor, Polidori stated that if a soil is made up of clay (34 %), silt (33 %) and sand (33 %), and thus classified, according to particle size distribution, as ‘clay with. . .’ but on the contrary such soil would be classified as silt on the plasticity chart based on its behavioral standpoint. This may be attributed to the fact that silt and sand components occur in roughly equal proportion in the fraction 2–425 μm unlike in the grain size distribution.

We also assume that the difference may also arise because of other factors. For instance, the nature of soil used to develop the modified plasticity chart may account for the differences. The lateritic soils considered in this study contain a significant amount of silt particle size. However, the modified plasticity chart was developed using a mixture of pure clay (kaolinite and montmorillonite) and fine silica sand. Therefore, the soil lacks silt particle size and the effect this particle size range have on the line that separates silt from clay (0.5C – line) on the modified plasticity chart could not be ascertained. In addition, the difference may also arise because of differences in fractions of soils used to designate a soil as coarse or fine grained (i.e., soil particles finer than the No. 200 sieve (0.075mm)) and fraction of soils used to evaluate the W_L_ and W_P_ of soils fractions termed as fine-grained soils (i.e., soil particles finer than No. 40 sieve (425 μm)).

Considering the mineral constituents of the rocks from which the lateritic soils were derived, one might argue that Polidori's modified plasticity chart may give the appropriate classification of the soils in cases where the lateritic soils plot in a zone that do not corresponds to their clay fractions. In such a situation, it is possible that the parent rock yields lateritic soils with high amount of sand and silt but with low amount clay that are increasingly expandable, which influence the properties and behavior of the soils.

From this review it is evident that the use of Polidori's modified plasticity chart gives a fair classification of lateritic soils based on soil fractions. However, the difficulties associated with the use of a classification scheme primarily designed for temperate and transported soils to classify residual soils must not be put aside. Vaughan et al. (1988) stated that classification systems developed using remolded soils usually produce inaccurate classification when used to classify residual soils because the properties of residual soils are influenced by in situ structures or structural characteristics of the parent rocks or properties developed because of prolonged weathering. Experience has shown that the use of tropical residual soils such as lateritic soils as engineering soils may not produce the same test results when assessed using standard laboratory test procedures mainly used for temperate soils. It has been found that the way iron and aluminum oxides occur (amorphous colloids or with aggregating effect on clay minerals) in tropical residual soils can suppress or contribute to their plasticity (Newill, 1961; Townsend et al., 1971). In addition, the clay contents and Atterberg limits of tropical soils are affected by method of pre-test drying (oven dried, air dried or tested from natural moisture content) adopted (Anon, 1990; Newill, 1961). Malomo et al. (1983) observed that drying usually decreases the W_L_ and W_P_ of oven-dried soils when compared to air-dried soils.

## 5 Activity of the lateritic soils

The behavior of soils is greatly affected by the amount and types of clay minerals that exist in such soil. Soils, which have the same clay percentage, may show a significant variation in Atterberg limits values because of the type of clay minerals. In addition, the type and amount of clay minerals in a soil may make soils, which have equal W_L_ and I_P_, have different engineering properties. Thus, Polidori (2009) stated that the Atterberg limits and amount of clay are not sufficient to classify, characterize and predict the behavior of fine grained soil. To separate the effect of the amount and types of clay minerals, Skempton in 1953 introduced the term “Activity”. Activity, according to Shakoor (2016), denotes the sensitivity of fine-gained soils to variations in moisture content. It is very suitable in determining the swelling behavior of a clayey soil. The activity of the clay (Table 2) in the lateritic soils’ samples (or part of the lateritic soils samples) presented by (Adeyemi, 1995; Adeyemi et al., 2015; Adeyemi and Oyeyemi, 2000; Adeyemi and Wahab, 2008) are active. On the other hand, clay minerals present in lateritic soil as reported by (Badmus, 2010; Ogunsanwo, 1986; Osinubi and Nwaiwu, 2006; Oyediran and Okosun, 2013) are inactive or normal clays.

**Table 2:** Skempton and polidori activity classification of the lateritic soils.

NO	ACTIVITY CLASSIFICATION	
	Skempton (1953)	Polidori (2009)
1	Inactive	CL
2	Inactive	CM
3	Inactive	CL
4	Inactive	CM
5	Inactive	CL
6	Inactive	CM
7	Inactive	CL
8	Normal	ML
9	Active	CL
10	Normal	MM
11	Normal	MM
12	Normal	CM
13	Active	CM
14	Active	MH
15	Active	CL
16	Active	MM
17	Active	CL
18	Active	CL
19	Active	CL
20	Inactive	CL
21	Active	CL
22	Normal	CL
23	Inactive	CL
24	Inactive	CL
25	Active	MM
26	Normal	MM
27	Normal	MH
28	Active	CL
29	Inactive	CL
30	Inactive	ML
31	Inactive	ML
32	Inactive	ML
33	Active	MM
34	Active	MM
35	Active	MM
36	Active	CM
37	Active	ML
38	Active	CL
39	Active	MM
40	Active	MH
41	Inactive	CL
42	Normal	CL
43	Inactive	CL
44	Normal	CL
45	Inactive	ML
46	Inactive	CL
47	Inactive	ML
48	Inactive	ML
49	Inactive	CL
50	Inactive	CL
51	Normal	ML
52	Normal	ML
53	Normal	MM
54	Normal	CL
55	Normal	ML
56	Normal	ML
57	Active	ML
58	Active	MM
59	Active	ML
60	Normal	CL
61	Inactive	MH
62	Inactive	MH
63	Inactive	ML
64	Normal	CL
65	Normal	MM
66	Active	MM
67	Active	ML
68	Normal	ML
69	Normal	ML
70	Normal	ML
71	Normal	ML
72	Active	CL
73	Normal	CL
74	Active	CL
75	Active	CL

1–7: Ogunsanwo (1986), 8–12: Ogunsanwo (1988), 13–24: Adeyemi (1995), 25–27: Ogunsanwo (1996), 28–29: Adeyemi and Oyeyemi (2000), 30–32: Osinubi and Nwaiwu (2006), 33–40: Adeyemi and Wahab (2008), 41–60: Badmus (2010), 61–63: Oyediran and Okosun (2013), 64–75: Adeyemi et al. (2015).

**Figure 6:**
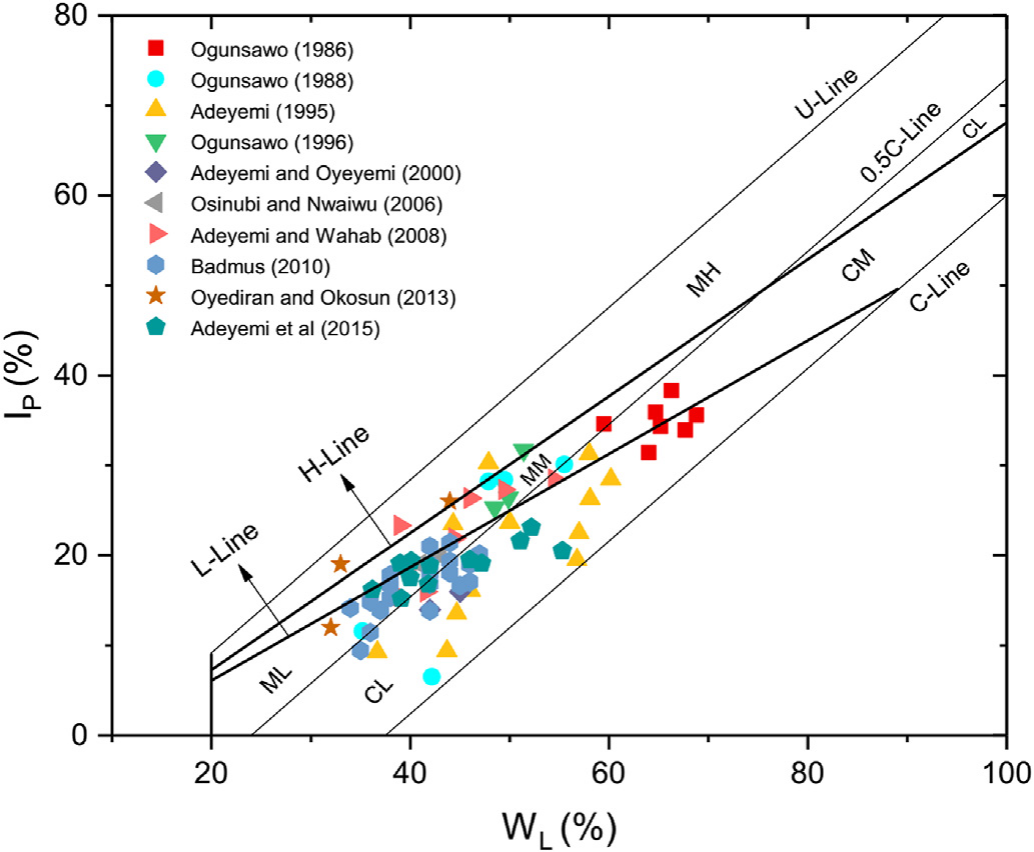
Polidori Activity chart of the lateritic soils. H-Line and L-line: lines on which IP vs WL values of inorganic soil with 1.0 and 0.5 values of activity, respectively, should lie; CL, CM, CH: groups of clays (CF ≥ 50 %) with low, medium and high activity, respectively; ML, MM, MH: groups of silts (CF > 50 %) with low, medium and high activity, respectively; O: organic soils; NPC: soil with non-platey clay minerals.

The classification of the lateritic soils using Polidori's activity chart is shown in Figure 6 and Table 2. The lateritic soils are classified as either clay with low activity (CL), clay with medium activity (CM), silt with low activity (ML), silt with medium activity (MM) or silt with high activity (MH) based on Polidori's activity chart. It is; however, observed that lateritic soils classified as silt with low plasticity, in Polidori's modified plasticity chart, are classified using Polidori's activity chart as either silt with low activity, silt with medium activity or silt with high activity (Tables 1 and 2). In addition, lateritic soils classified as silt with high plasticity are classified as silt with high activity using the new activity chart. On the other hand, lateritic soils designated as clay with high plasticity are classified on the new activity chart, as clay with low or medium activity. Lateritic soils classified as clay with low plasticity are classified as clay with low activity using the new activity chart. The fact that soils in the same plasticity zone lie in different activity zones indicate that classification in the plasticity charts is based on liquid limit where the expandability of clay minerals in the soil is ignored.

Most lateritic soils classified as silt in Polidori's modified plasticity chart and with active activity according to Skempton activity (Tables 1 and 2) lie in either medium or low activity silt zone on the new activity chart. Surprisingly, on the new activity chart, lateritic soils classified as silt on the modified plasticity chart and with either normal or inactive activity according to Skempton activity classification can lie in either the high or medium or low activity silt zone. In addition, most lateritic soils classified as clay on the Polidori's modified plasticity chart and with active activity according to Skempton activity (Tables 1 and 2) lie in the low or medium activity clay zone on the new activity chart. This is the same with clayey soils with normal and inactive activity. The difference in the two-activity classification systems may be due to the way the authors produced the charts.

## 6 Conclusions

This study investigated the use of modified plasticity and activity charts proposed by Polidori in 2007 on selected residual lateritic soils from different rock types in Nigeria. Casagrande's plasticity chart shows that the lateritic soils are mostly inorganic soil and plot above the A-line in the zone designated as clay and classified as either CL or CH.

The modified plasticity chart also shows that the lateritic soils are inorganic soils. The classifications obtained from the new Polidori plasticity chart are mainly in agreement with the main soil fraction or component of the soils except in few cases where the lateritic soils plot in clay or silt zone when their clay contents are less or greater than 50 %, respectively. Lateritic soils classified as CH or CL on the Casagrande's plasticity chart are classified as MH or ML on the modified Poridori's plasticity chart and vice versa. Polidori's activity chart shows that the lateritic soil that lies in the same plastic zone on the two plasticity charts may behave differently because of the different expandability of the clay mineral present in the soils.

Polidori's modified plasticity chart seems to give a fair classification of the lateritic soils but similar to Casagrande's plasticity chart it was developed based on the properties of remolded soils (in this case mixture of pure clays and fine sand) without considering the in-situ structures that residual lateritic soils may acquire from the parent rock or developed as a result of prolonged weathering.

## Declarations

### Author contribution statement

Lekan Olatayo Afolagboye: Conceived and designed the experiments; Performed the experiments; Analyzed and interpreted the data; Contributed reagents, materials, analysis tools or data; Wrote the paper.

Abel Ojo Talabi: Analyzed and interpreted the data; Wrote the paper.

Olubunmi Oluwadare Owoyemi: Performed the experiments; Contributed reagents, materials, analysis tools or data.

### Funding statement

This research did not receive any specific grant from funding agencies in the public, commercial, or not-for-profit sectors.

### Data availability statement

Data included in article/supplementary material/referenced in article.

### Declaration of interests statement

The authors declare no conflict of interest.

### Additional information

No additional information is available for this paper.

## Appendix A Supplementary data

S1 S1
