# Supplementary material for: The use of Polidori's plasticity and activity charts in classifying some residual lateritic soils from Nigeria
Source: Heliyon. 2021 Aug 3;7(8):e07713. doi: 10.1016/j.heliyon.2021.e07713 (PMC8350542; doi:10.1016/j.heliyon.2021.e07713)
Supplement: S1 [file mmc1.docx]

Supplementary File

Table S1: Properties and the classification of the lateritic soils

| No | Parent Rock | W_L_ (%) | I_p_  (%) | A | Grain Size | | | | Plasticity Classification | | Activity Classification | | Clay Mineralogy | Method of testing | | Reference |
| --- | --- | --- | --- | --- | --- | --- | --- | --- | --- | --- | --- | --- | --- | --- | --- | --- |
|  |  |  |  |  | Gravel (%) | Sand (%) | Silt (%) | Clay (%) | Casagrande (1948) | Polidori (2007) | Skempton (1953) | Polidori (2009) |  |  |  |  |
| 1 | Amphibolite | 65.2 | 34.3 | 0.54 | - | 13 | 24 | 63 | CH | CH | Inactive | CL | Kaolinite and goethite | BS 1377 | Ogunsawo (1986) | |
| 2 | Amphibolite | 59.5 | 34.6 | 0.61 | - | 14 | 29 | 57 | CH | CH | Inactive | CM |  |  | Ogunsawo (1986) | |
| 3 | Amphibolite | 64 | 31.4 | 0.52 | - | 18 | 22 | 60 | MH | CH | Inactive | CL |  |  | Ogunsawo (1986) | |
| 4 | Amphibolite | 64.7 | 35.9 | 0.58 | - | 13 | 25 | 62 | CH | CH | Inactive | CM |  |  | Ogunsawo (1986) | |
| 5 | Amphibolite | 68.8 | 35.6 | 0.59 | - | 15 | 25 | 60 | MH | CH | Inactive | CL |  |  | Ogunsawo (1986) | |
| 6 | Amphibolite | 66.3 | 38.3 | 0.67 | - | 18 | 22 | 60 | CH | CH | Inactive | CM |  |  | Ogunsawo (1986) | |
| 7 | Amphibolite | 67.7 | 33.9 | 0.57 | - | 18 | 25 | 57 | MH | CH | Inactive | CL |  |  | Ogunsawo (1986) | |
| 8 | Quartz Schist | 35.2 | 11.6 | 0.97 | 2 | 46 | 40 | 12 | CL | ML | Normal | ML | Kaolinite, Goethite, Muscovite, Quartz, Feldspar | DIN 18123 and DIN 18122/1 | Ogunsawo (1988) | |
| 9 | Mica Schist | 42.2 | 6.5 | 1.3 | 0 | 35 | 60 | 5 | ML | CL | Active | CL |  |  | Ogunsawo (1988) | |
| 10 | Coastal Plain Sand | 49.5 | 28.4 | 0.81 | 2 | 48 | 15 | 35 | CL | ML | Normal | MM |  |  | Ogunsawo (1988) | |
| 11 | Granite Gneiss | 47.9 | 28.2 | 0.97 | 12 | 35 | 24 | 29 | CL | ML | Normal | MM |  |  | Ogunsawo (1988) | |
| 12 | Amphibolite | 55.5 | 30.1 | 0.79 | 3 | 23 | 36 | 38 | CH | CH | Normal | CM |  |  | Ogunsawo (1988) | |
| 13 | Sand stone | 58 | 31.3 | 3.13 | 18 | 40 | 32 | 10 | CH | CH | Active | CM | NM | BS 1377 | Adeyemi (1995) | |
| 14 | Sand stone | 47.9 | 30.3 | 3.03 | 7 | 45 | 38 | 10 | CL | ML | Active | MH |  |  | Adeyemi (1995) | |
| 15 | Sand stone | 50 | 23.7 | 2.63 | 3 | 49 | 39 | 9 | CL | CL | Active | CL |  |  | Adeyemi (1995) | |
| 16 | Sand stone | 44.3 | 23.5 | 3.36 | 10 | 42 | 41 | 7 | CL | ML | Active | MM |  |  | Adeyemi (1995) | |
| 17 | Migmatite Gneiss | 60.2 | 28.5 | 2.38 | 4 | 22 | 62 | 12 | MH | CH | Active | CL |  |  | Adeyemi (1995) | |
| 18 | Migmatite Gneiss | 57 | 22.5 | 1.73 | 3 | 29 | 55 | 13 | MH | CH | Active | CL |  |  | Adeyemi (1995) | |
| 19 | Migmatite Gneiss | 58.1 | 26.3 | 2.02 | 1 | 37 | 49 | 13 | MH | CH | Active | CL |  |  | Adeyemi (1995) | |
| 20 | Migmatite Gneiss | 56.8 | 19.5 | 0.7 | 6 | 34 | 32 | 28 | MH | CH | Inactive | CL |  |  | Adeyemi (1995) | |
| 21 | Quartz Schist | 36.7 | 9.3 | 1.86 | 53 | 29 | 13 | 5 | ML | CL | Active | CL |  |  | Adeyemi (1995) | |
| 22 | Quartz Schist | 46.1 | 16.1 | 0.89 | 12 | 40 | 30 | 18 | ML | CL | Normal | CL |  |  | Adeyemi (1995) | |
| 23 | Quartz Schist | 43.7 | 9.4 | 0.45 | 8 | 44 | 27 | 21 | ML | CL | Inactive | CL |  |  | Adeyemi (1995) | |
| 24 | Quartz Schist | 44.7 | 13.6 | 0.65 | 7 | 44 | 28 | 21 | ML | CL | Inactive | CL |  |  | Adeyemi (1995) | |
| 25 | Benin sand | 49.9 | 26.4 | 1.26 | 3 | 57 | 19 | 21 | CL | ML | Active | MM | NM | ONORM B 4411,4412,4413,4418 and 4422 | Ogunsanwo (1996) | |
| 26 | Migmatite | 48.5 | 25.3 | 1.2 | 9 | 48 | 22 | 21 | CL | ML | Normal | MM |  |  | Ogunsawo (1996) | |
| 27 | Granite Gneiss | 51.4 | 31.7 | 0.93 | 1 | 20 | 45 | 34 | CH | MH | Normal | MH |  |  | Ogunsawo (1996) | |
| 28 | Granitic gneiss | 45 | 16 | 2.29 | 12.5 | 48 | 32.5 | 7 | ML | CL | Active | CL | NM | BS 1377 | Adeyemi and Oyeyemi (2000) | |
| 29 | Porphyritic granite | 42 | 14 | 0.7 | 13 | 35 | 32 | 20 | ML | CL | Inactive | CL |  |  | Adeyemi and Oyeyemi (2000) | |
| 30 | NM | 43 | 20 | 0.61 | 0.3 | 37 | 29.7 | 33 | CL | ML | Inactive | ML | NM | BS 1377 | Osinubi and Nwaiwu (2006) | |
| 31 | NM | 41 | 19 | 0.58 | 0.6 | 29 | 37.4 | 33 | CL | ML | Inactive | ML |  |  | Osinubi and Nwaiwu (2006) | |
| 32 | NM | 40 | 18 | 0.55 | 3.8 | 37 | 26.2 | 33 | CL | ML | Inactive | ML |  |  | Osinubi and Nwaiwu (2006) | |
| 33 | Migmatite Gneiss pegmatite | 46 | 26.4 | 2.03 | 19 | 26.2 | 41.8 | 13 | CL | ML | Active | MM | NM | BS 1377 | Adeyemi and Wahab (2008) | |
| 34 | Migmatite Gneiss pegmatite | 46 | 26.3 | 2.02 | 18 | 29.2 | 39.8 | 13 | CL | ML | Active | MM |  |  | Adeyemi and Wahab (2008) | |
| 35 | Migmatite Gneiss pegmatite | 49.5 | 27.3 | 4.96 | 25 | 22.7 | 46.8 | 5.5 | CL | ML | Active | MM |  |  | Adeyemi and Wahab (2008) | |
| 36 | Migmatite Gneiss pegmatite | 54.5 | 28.5 | 4.75 | 24 | 23 | 47 | 6 | CH | CH | Active | CM |  |  | Adeyemi and Wahab (2008) | |
| 37 | Migmatite Gneiss pegmatite | 39 | 15.5 | 1.94 | 20 | 22.5 | 49.5 | 8 | CL | ML | Active | ML |  |  | Adeyemi and Wahab (2008) | |
| 38 | Migmatite Gneiss pegmatite | 41.6 | 16 | 2 | 18 | 24.9 | 49.1 | 8 | CL | CL | Active | CL |  |  | Adeyemi and Wahab (2008) | |
| 39 | Migmatite Gneiss pegmatite | 44.5 | 21.9 | 7.3 | 23 | 20.8 | 53.2 | 3 | CL | ML | Active | MM |  |  | Adeyemi and Wahab (2008) | |
| 40 | Migmatite Gneiss pegmatite | 39 | 23.3 | 7.77 | 22 | 20.8 | 54.2 | 3 | CL | ML | Active | MH |  |  | Adeyemi and Wahab (2008) | |
| 41 | Quartz schist | 46 | 17.08 | 0.55 | 3 | 42 | 24 | 31 | ML | CL | Inactive | CL | NM | NM | Badmus (2010) | |
| 42 | Quartz schist | 45 | 16.66 | 0.98 | 4 | 50 | 29 | 17 | ML | CL | Normal | CL |  |  | Badmus (2010) | |
| 43 | Quartz schist | 46 | 18.95 | 0.59 | 6 | 41 | 21 | 32 | ML | CL | Inactive | CL |  |  | Badmus (2010) | |
| 44 | Quartz schist | 44 | 19.39 | 0.84 | 5 | 48 | 24 | 23 | CL | CL | Normal | CL |  |  | Badmus (2010) | |
| 45 | Quartz schist | 37 | 13.89 | 0.66 | 6 | 49 | 24 | 21 | CL | ML | Inactive | ML |  |  | Badmus (2010) | |
| 46 | Quartz schist | 42 | 13.87 | 0.63 | 6 | 48 | 24 | 22 | ML | CL | Inactive | CL |  |  | Badmus (2010) | |
| 47 | Quartz schist | 38 | 15.34 | 0.61 | 8 | 52 | 15 | 25 | CL | ML | Inactive | ML |  |  | Badmus (2010) | |
| 48 | Quartz schist | 38 | 16.7 | 0.67 | 14 | 41 | 20 | 25 | CL | ML | Inactive | ML |  |  | Badmus (2010) | |
| 49 | Quartz schist | 36 | 11.47 | 0.52 | 6 | 48 | 24 | 22 | ML | CL | Inactive | CL |  |  | Badmus (2010) | |
| 50 | Quartz schist | 35 | 9.47 | 0.53 | 8 | 47 | 27 | 18 | ML | CL | Inactive | CL |  |  | Badmus (2010) | |
| 51 | Granite | 44 | 21.4 | 0.86 | 4 | 49 | 22 | 25 | CL | ML | Normal | ML |  |  | Badmus (2010) | |
| 52 | Granite | 42 | 16.85 | 1.12 | 2 | 57 | 26 | 15 | CL | ML | Normal | ML |  |  | Badmus (2010) | |
| 53 | Granite | 42 | 21 | 1.05 | 4 | 51 | 25 | 20 | CL | ML | Normal | MM |  |  | Badmus (2010) | |
| 54 | Granite | 44 | 18.06 | 0.82 | 7 | 51 | 20 | 22 | CL | CL | Normal | CL |  |  | Badmus (2010) | |
| 55 | Granite | 34 | 14.19 | 0.89 | 10 | 57 | 17 | 16 | CL | ML | Normal | ML |  |  | Badmus (2010) | |
| 56 | Granite | 36 | 14.87 | 0.87 | 10 | 50 | 23 | 17 | CL | ML | Normal | ML |  |  | Badmus (2010) | |
| 57 | Granite | 42 | 19 | 1.27 | 3 | 60 | 22 | 15 | CL | ML | Active | ML |  |  | Badmus (2010) | |
| 58 | Granite | 38 | 17.82 | 1.37 | 2 | 62 | 23 | 13 | CL | ML | Active | MM |  |  | Badmus (2010) | |
| 59 | Granite | 42 | 18.01 | 1.29 | 3 | 67 | 16 | 14 | CL | ML | Active | ML |  |  | Badmus (2010) | |
| 60 | Granite | 47 | 20.16 | 1.06 | 8 | 57 | 16 | 19 | CL | CL | Normal | CL |  |  | Badmus (2010) | |
| 61 | Sedimentary | 33 | 19 | 0.33 | 8 | 27 | 57 | 8 | CL | ML | Inactive | MH | Quartz, Kaolinite, Labradorite,  Haematite | BS 1377 | Oyediran and Okosun (2013) | |
| 62 | Sedimentary | 44 | 26 | 0.58 | 8 | 35 | 45 | 12 | CL | ML | Inactive | MH |  |  | Oyediran and Okosun (2013) | |
| 63 | Sedimentary | 32 | 12 | 0.33 | 14 | 43 | 36 | 7 | CL | ML | Inactive | ML |  |  | Oyediran and Okosun (2013) | |
| 64 | Coastal Plain Sand | 47.2 | 19.1 | 1.12 | 3 | 37 | 46 | 17 | ML | CL | Normal | CL | NM | BS 1377 | Adeyemi et al (2015) | |
| 65 | Coastal Plain Sand | 40.1 | 19.4 | 1.21 | 2 | 35 | 47 | 16 | CL | ML | Normal | MM |  |  | Adeyemi et al (2015) | |
| 66 | Coastal Plain Sand | 39 | 19.1 | 1.27 | 3 | 37 | 45 | 15 | CL | ML | Active | MM |  |  | Adeyemi et al (2015) | |
| 67 | Coastal Plain Sand | 42 | 18.9 | 1.26 | 2 | 36 | 47 | 15 | CL | ML | Active | ML |  |  | Adeyemi et al (2015) | |
| 68 | Coastal Plain Sand | 36.2 | 16.2 | 1.01 | 3 | 37 | 44 | 16 | CL | ML | Normal | ML |  |  | Adeyemi et al (2015) | |
| 69 | Coastal Plain Sand | 39.1 | 15.2 | 0.89 | 3 | 35 | 45 | 17 | CL | ML | Normal | ML |  |  | Adeyemi et al (2015) | |
| 70 | Coastal Plain Sand | 41.8 | 16.8 | 1.05 | 3 | 36 | 45 | 16 | CL | ML | Normal | ML |  |  | Adeyemi et al (2015) | |
| 71 | Coastal Plain Sand | 40 | 17.5 | 0.97 | 2 | 38 | 42 | 18 | CL | ML | Normal | ML |  |  | Adeyemi et al (2015) | |
| 72 | Coastal Plain Sand | 52.2 | 23.1 | 1.44 | 3 | 32 | 49 | 16 | MH | CH | Active | CL |  |  | Adeyemi et al (2015) | |
| 73 | Coastal Plain Sand | 55.3 | 20.5 | 1.14 | 3 | 31 | 48 | 18 | MH | CH | Normal | CL |  |  | Adeyemi et al (2015) | |
| 74 | Coastal Plain Sand | 51.1 | 21.6 | 1.27 | 2 | 36 | 45 | 17 | MH | CH | Active | CL |  |  | Adeyemi et al (2015) | |
| 75 | Coastal Plain Sand | 46 | 19.5 | 1.3 | 2 | 33 | 50 | 15 | CL | CL | Active | CL |  |  | Adeyemi et al (2015) | |

NM not mentioned, CL: Inorganic clays of low plasticity, CH: Inorganic clays of high plasticity; ML: Inorganic silts of low compressibility; MH: Inorganic silts of high compressibility
